# Characterization of Quorum Sensing and Quorum Quenching Soil Bacteria Isolated from Malaysian Tropical Montane Forest

**DOI:** 10.3390/s120404846

**Published:** 2012-04-13

**Authors:** Teik-Min Chong, Chong-Lek Koh, Choon-Kook Sam, Yeun-Mun Choo, Wai-Fong Yin, Kok-Gan Chan

**Affiliations:** 1 Division of Genetics and Molecular Biology, Institute of Biological Sciences, Faculty of Science, University of Malaya, 50603 Kuala Lumpur, Malaysia; E-Mails: cluster1986@hotmail.com (T.-M.C.); yinwaifong@yahoo.com (W.-F.Y.); 2 Natural Sciences and Science Education AG, National Institute of Education, Nanyang Technological University, 1 Nanyang Walk, 637616, Singapore; E-Mails: chonglek.koh@nie.edu.sg (C.-L.K.); choonkook.sam@nie.edu.sg (C.-K.S.); 3 Department of Chemistry, Faculty of Science, University of Malaya, 50603 Kuala Lumpur, Malaysia; E-Mail: ymchoo@um.edu.my

**Keywords:** *Arthrobacter*, *Bacillus*, liquid chromatography mass spectrometry (LC-MS), *N*-acylhomoserine lactone, *N*-dodecanoyl-L-homoserine lactone, *p*-coumaroylhomoserine lactone, *Pseudomonas frederiksbergensis*, quorum quenching, quorum sensing, rapid resolution liquid chromatography (RRLC)

## Abstract

We report the production and degradation of quorum sensing *N*-acyl-homoserine lactones by bacteria isolated from Malaysian montane forest soil. Phylogenetic analysis indicated that these isolates clustered closely to the genera of *Arthrobacter, Bacillus* and *Pseudomonas*. Quorum quenching activity was detected in six isolates of these three genera by using a series of bioassays and rapid resolution liquid chromatography analysis. Biosensor screening and high resolution liquid chromatography-mass spectrometry analysis revealed the production of *N*-dodecanoyl-L-homoserine lactone (C12-HSL) by *Pseudomonas frederiksbergensis* (isolate BT9). In addition to degradation of a wide range of *N*-acyl-homoserine lactones, *Arthrobacter* and *Pseudomonas* spp. also degraded *p*-coumaroyl-homoserine lactone. To the best of our knowledge, this is the first documentation of *Arthrobacter* and *Pseudomonas* spp. capable of degrading *p*-coumaroyl-homoserine lactone and the production of C12-HSL by *P. frederiksbergensis*.

## 1. Introduction

“Quorum sensing” (QS) describes the bacterial cell-to-cell communication mechanism through the binding of diffusible signals to their cognate receptor proteins, thereby regulating gene expression in response to bacterial cell density [1,2]. These signals, known as autoinducers, are synthesized at specific stages of growth or in response to changes in the environment and induce a concerted response once a critical concentration of signaling molecules is reached [1]. When a bacterial population reaches a threshold cell density, the concentration of the QS signal is sufficient to induce gene expression either directly by interacting with a transcriptional regulator or indirectly by activating a signaling cascade [3].

Arguably, one of the most well studied QS signals is *N*-acylhomoserine lactone (AHL) [4]. AHL signaling molecules are highly conserved as they have the same homoserine lactone moiety, but differ in the length and structure of the acyl side chain. The *N*-acylated side chain consists of fatty acids that vary in chain length (ranging from 4–18 carbons), degree of saturation, and the presence of a hydroxy-, oxo- or no substituent at the C3 position [5]. AHL molecules are synthesized by LuxI synthase using *S*-adenosylmethionine and an intermediate of fatty acid biosynthesis as substrates. The generated AHL molecules will then bind to LuxR protein as a receptor of AHL followed by subsequent regulation of downstream gene expressions. Each of the LuxR-type proteins is highly selective for its cognate AHL signal molecules [6].

Although different target genes are regulated by AHLs, the basic mechanisms of AHL biosynthesis and gene regulation seem to be conserved in different QS bacterial species [7]. Many of these bacteria possess a QS mechanism with the LuxI/LuxR signal-response circuit as the backbone with additional complexity. For instance, the phytopathogen *Agrobacterium tumefaciens*, which is responsible for crown gall tumors in plants, uses the tumour-derived opines and the transcriptional factor OccR or AccR to regulate the expression of the LuxR homologue TraR [8,9]. The opportunistic pathogen *Pseudomonas aeruginosa* employs two pairs of LuxI/LuxR like systems, namely LasI/R and RhlI/R, that function in tandem to regulate the expression of virulence factors and biofilm formation [10]. Another group of homoserine lactone known as *p*-coumaroylhomoserine lactone (pC-HSL) has been discovered to be produced by the photosynthetic bacterium *Rhodopseudomonas palustris*. Synthesis of pC-HSL molecules were catalyzed by RpaI, a LuxI homolog, which uses environmental *p*-coumaric acid rather than intracellular fatty acids as a pC-HSL precursor [11].

QS has been employed by many bacteria to gain maximal competition advantages; hence, various organisms use quorum quenching (QQ) to countermeasure the benefits of QS [12]. This QQ mechanism that plays important roles in microbe-microbe and pathogen-host interactions is widely distributed in prokaryotes and eukaryotes [13].

For instance, inhibition of QS can be achieved by destabilizing the LuxR family protein receptors for AHL signal molecules or by degrading AHLs by lactonases and acylases [12,14]. Acylase hydrolyzes the amide bond of AHL releasing the fatty acid and homoserine lactone (HSL) [15]. The fatty acid released is utilized as an energy source whereas the HSL released can be exploited as nitrogen source through mineralization of the lactone ring [13,16]. On the other hand, lactonase hydrolyses the homoserine lactone ring producing acyl homoserine [16]. The third enzyme, AHL-oxidoreductase, originally identified in *Rhodococcus erythropolis*, reduces the keto-group of 3-oxo-AHLs to the corresponding 3-hydroxy derivative. However, this AHL-oxidoreductase is inactive against hydroxylated and fully reduced AHLs [17]. In addition, there are also several small molecules that are capable of inhibiting QS. These molecules are either structural mimics of QS signals or enzyme inhibitors and act by interfering with the corresponding signal binding to the receptor or decreasing the receptor concentration, therefore disrupting the QS mechanism [18,19].

Because bacterial community cooperation of QS enhances the effectiveness of many processes especially virulence determinants [4,20], therefore, QQ is of significant interest as a novel anti-infective therapy [21]. In view of this, we investigated the presence of QS and QQ bacteria in a Malaysian tropical montane forest soil sample. Here, we report the isolation of *Arthrobacter, Bacillus* and *Pseudomonas* strains from it.

## 2. Experimental Section

### 2.1. Soil Sampling and Isolation of Bacteria

Soil sampling was carried out in 2010 in a site several meters away from a tea plantation in Cameron Highlands, Malaysia. The GPS coordinates for the site were N04°32″707′ E101°25″275′, at an elevation of 1,206 m above sea level. Soil was collected at a depth of 5 cm below the soil surface into a sterile 50-mL centrifuge tube and immediately processed upon returning to the laboratory. To process the soil, stones and roots were removed manually with sterile spatula. A soil suspension was prepared by mixing the soil sample (1 g) with PBS buffer (100 mM, pH 6.5; 5 mL) by vigorous vortex. The soil suspension was serially diluted and spread onto LB agar. Bacteria with observable different morphology were isolated after incubation for 24 h at 28 °C. Pure colony was obtained with several passages on LB agar.

### 2.2. Bacterial Strains and Culture Conditions

Table 1 lists the bacterial strains used. For AHL degrading bioassays, *Bacillus cereus* was used as the positive control whereas *Escherichia coli* TOP10 cells were used as negative control [22]. AHL production screening involves *Erwinia carotovora* Attn as positive control and *E. carotovora* A20 served as negative control (defective for AHL production). *Chromobacterium violaceum* CV026 served as AHL biosensor with formation of purple violacein pigment in presence of short chain exogenous AHLs molecules [23]. *E. coli* TOP10 cell was also used as host for cloning. Subsequent growth of the sampled soil isolates, *E. carotovora* and *B. cereus* were carried out at 28 °C in LB media whereas Except for *E. coli* TOP10, which was cultured at 37 °C, all other bacterial strains were cultured at 28 °C.

### 2.3. Strain Identification

Bacterial 16S rDNA genes were PCR-amplified with the forward primer 27F (5′-AGAGTTTGATC MTGGCTCAG-3′), the reverse primer 1525R (5′-AAGGAGGTGWTCCARCC-3′) [24], and genomic DNA extracted using the QIAamp DNA Mini Kit (Qiagen Inc.). The PCR cycles consisted of an initial denaturation at 94 °C for 5 min, followed by 30 cycles at 94 °C for 30 s, annealing at 63 °C for 30 s and extension at 72 °C for 1 min 30 s, and a final extension at 72 °C for 5 min. Cloning and sequencing of the PCR products were carried out as described previously [24]. Gene sequences were compared with GenBank databases using the BLASTN program followed by sequence alignment and phylogenetic analyses using the Molecular Evolutionary Genetic Analysis (MEGA) version 5 [25].

### 2.4. Screening of AHL Degradation

Overnight grown bacterial cells were harvested by centrifugation and whole-cell AHL inactivation assay was performed as described previously [26]. Briefly, the cell pellets were washed twice and resuspended in PBS (100 mM, pH 6.5). Known amounts of selected synthetic AHLs (Sigma-Aldrich) were dispensed into microcentrifuge tubes and dried by evaporation, cells suspension was added to rehydrate the AHL to the final concentration of 1 μM. The mixtures were then incubated at 28 °C with shaking at 220 rpm for 0 h and 24 h for CV026 bioassays, and for 0 h, 3 h and 24 h for rapid resolution liquid chromatography (RRLC) analysis. All reactions were stopped by heat inactivation at 95 °C. For the detection of AHL degradation, 10 μL of reaction mixture was spotted onto sterile paper discs placed on a CV026 lawn and incubated overnight. Decreased violacein (purple zone) indicated AHL degradation. AHL inactivation assays involving incubation buffer (PBS buffer) and *B. cereus* served as negative and positive controls, respectively.

### 2.5. RRLC Analysis

Sample preparation for RRLC analysis was performed similar to that for the whole-cell AHL inactivation assay as described above with the exception that the final concentration of AHL was 50 μM after rehydration with a cell suspension. Residual AHL was extracted twice using ethyl acetate, followed by evaporation of the extract to dryness. Extracted AHL was resuspended in 100 μL of acetonitrile before RRLC analysis. The Agilent Technologies 1200 series RRLC system described by Wong [27] was used to analyze AHL degradation. AHL samples were separated in a Agilent Poroshell 120 EC-C18 column (4.6 mm × 100mm, 2.7 μm particle size) with the elution procedure consisting of an isocratic profile of acetonitrile/water (35:65, v/v) for short chain AHLs and acetonitrile/water (65:35, v/v) for long chain AHLs. A constant flow rate of 0.7 mL/min was applied and AHL detection was carried out at 210 nm with the exception of pC-HSL which was monitored at 306 nm [11]. Known amounts of synthetic AHLs or pC-HSL were also loaded as standards. AHL incubated with *E. coli* TOP10 cells or PBS served as a negative control.

### 2.6. Detection of AHL Production

Preliminary screening for AHL production by the soil bacterial isolates was by cross-streaking them on agar plates against the biosensor CV026. Purple pigmentation indicated presence of short chain AHLs. *E. carotovora* Attn was included as a positive control for AHL production, whereas *E. carotovora* A20 served as a negative control.

### 2.7. AHL Extraction

Bacteria were cultured in LB broth buffered to pH 6.5 with 50 mM of MOPS (100 mM, pH 6.5) and incubated overnight with shaking at 28 °C. The spent culture supernatant was extracted twice by addition of an equal volume of ethyl acetate followed by vortex mixing for 1 min. The organic solvent was dried in fume hood. The dried extracts were resuspended in 1 mL of ethyl acetate and dried again in fume hood. Finally, 200 μL of acetonitrile (HPLC grade) was added and vortexed for 3 min to dissolve the extracted AHLs. The mixture was left overnight at room temperature and centrifuged at 12,000 rpm for 10 min to remove any insoluble residue. Aliquots (75 μL) of the extract were withdrawn from the top layer and placed in sample vials for mass spectrometry analysis.

### 2.8. Identification of AHL by Mass Spectrometry (MS)

MS analysis of AHL was performed as described previously [27]. Agilent RRLC 1200 system was employed as the LC delivery system with the use of Agilent ZORBAX Rapid Resolution HT column (2.1 mm × 100 mm, 1.8 μm particle size). Analysis was carried out using a flow rate of 0.3 mL/min at 60 °C and the injection volume was 20 μL. Mobile phases A and B were 0.1% v/v formic acid in water and 0.1% v/v formic acid in acetonitrile, respectively. The gradient profiles were as follows (time: mobile phase A: mobile phase B): 0 min: 60:40, 5 min: 20:80, 7 and 10 min: 5:95, and 11 and 13 min: 60:40. The high-resolution electrospray ionization mass spectrometry (ESI-MS) was performed with the Agilent 6500 Q-TOF LC/MS system. The MS experiment was performed in the ESI-positive mode. The probe capillary voltage was set at 3 kV, desolvation temperature at 350 °C, sheath gas at 11 mL/h, and nebulizer pressure at 50 psi. The Agilent MassHunter software was used for the MS data analysis.

## 3. Results and Discussion

### 3.1. Isolation and Identification of Strains

This study entailed the detection of AHL production and degradation by soil bacteria isolated from a tropical montane area where the mean temperature is approximately 18 °C throughout the year [28]. The soil sample was collected at a site closed to a tea plantation where the biodiversity of the rhizosphere may have been altered due the application of pesticides or inorganic fertilizers in the tea estate, creating a selective environment favoring the growth of certain groups of microorganisms.

Nine bacteria isolated from the montane soil sample were selected for 16S rDNA identification. The isolates belonged to *Arthrobacter* sp., *Bacillus cereus* and *Pseudomonas frederiksbergensis* (Table 2). All strains shared 99% similarity in the BLAST search. Members of *Pseudomonas, Bacillus* and *Arthrobacter* genera are well known for their ubiquity in soil environment and resistance against chemicals [4,29,30].

### 3.2. Degradation of AHLs

**Figure 1.**
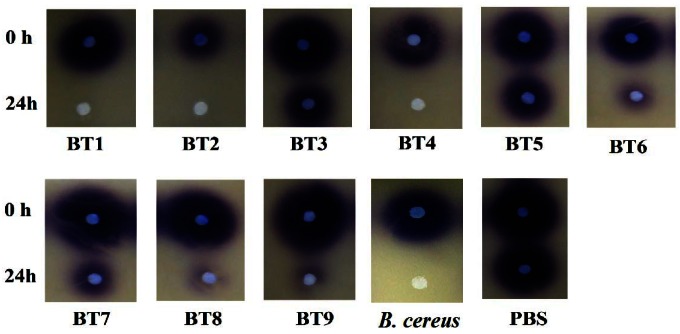
Detection of QQ activities using CV026 overlay. C6-HSL was incubated with bacterial cell suspensions for 0 h and 24 h. Positive result in QQ activity was depicted by the reduction or abolishment of purple pigments after 24 h of incubation. The positive and negative controls were *B. cereus* and PBS, respectively. See Table 2 for strains names.

**Figure 2.**
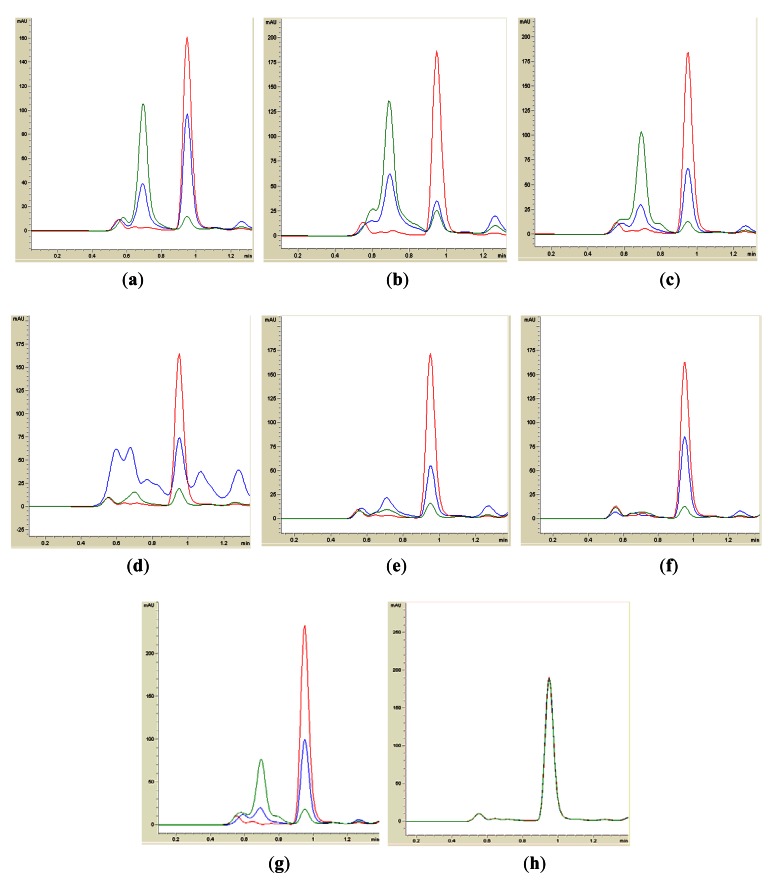
RRLC analysis of C10-HSL degradation. Residual C10-HSL (with elution time of 0.95 ± 0.02 min), after degradation for 0 h (red), 3 h (blue) and 24 h (green), was monitored at 210 nm. Degradation of C10-HSL was depicted by the reduction of milli-absorbance unit (mAU) in the chromatogram. Samples containing BT1 (**a**), BT2 (**b**), BT4 (**c**), BT6 (**d**), BT8 (**e**), BT9 (**f**) and *B. cereus* (**g**) showed significant degradation of C10-HSL. PBS buffer (**h**) was used as the negative control for the analysis.

**Figure 3.**
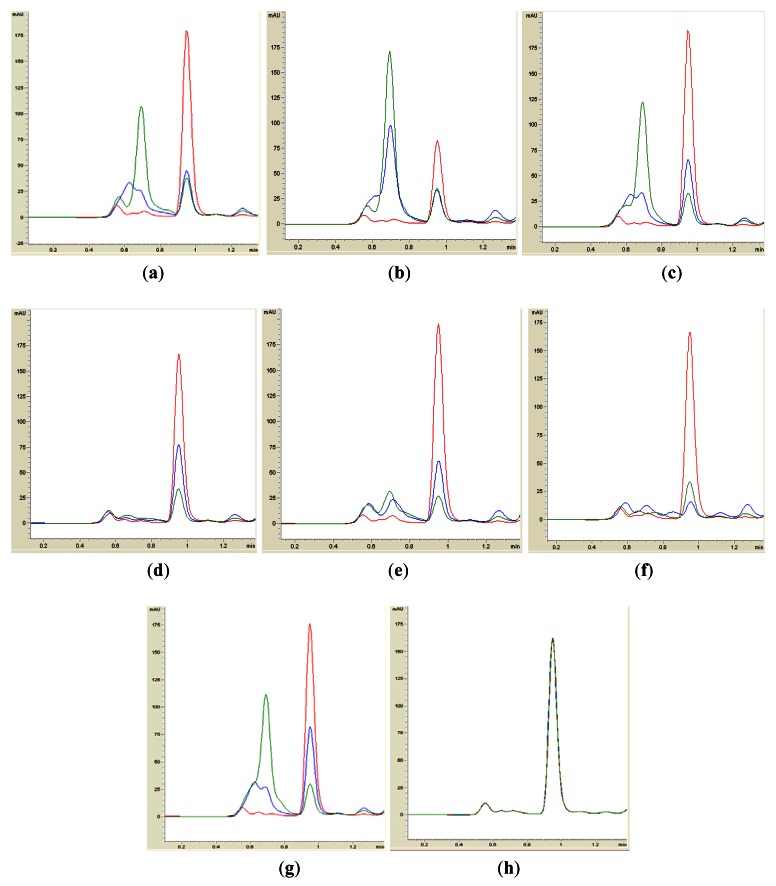
RRLC analysis of 3-oxo-C12-HSL degradation. Residual 3-oxo-C12-HSL (with elution time of 0.95 ± 0.02 min), after degradation for 0 h (red), 3 h (blue) and 24 h (green), was monitored and degradation of 3-oxo-C12-HSL was observed for all bacteria tested, such as BT1 (**a**), BT2 (**b**), BT4 (**c**), BT6 (**d**), BT8 (**e**), BT9 (**f**) and *B. cereus* (**g**). PBS solution (**h**) was the negative control for the analysis.

**Figure 4.**
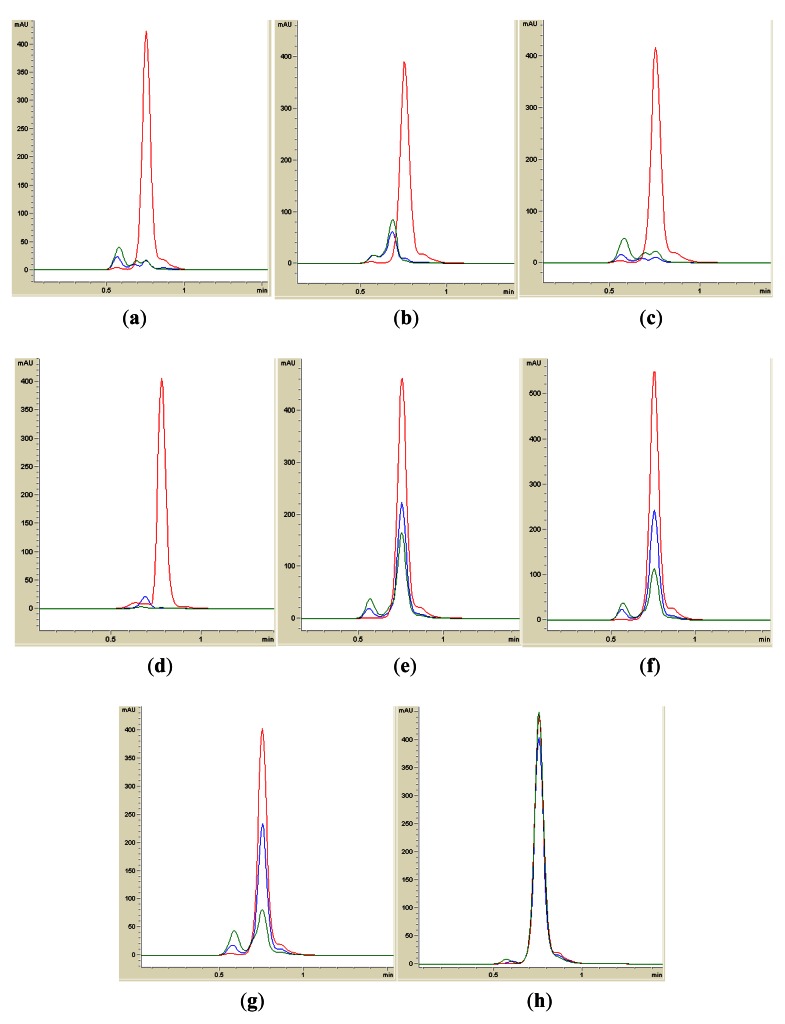
RRLC analysis of pC-HSL degradation. Residual pC-HSL (with elution time of 0.75 ± 0.01 min), after degradation for 0 h (red), 12 h (blue) and 24 h (green), was monitored at 306 nm. Degradation of pC-HSL was depicted by the reduction of milli-absorbance unit (mAU) in the chromatogram. Samples containing BT1 (**a**), BT2 (**b**), BT4 (**c**), BT6 (**d**), BT8 (**e**), BT9 (**f**) and *B. cereus* (**g**) showed degradation of pC-HSL. *E. coli* TOP10 (**h**) was used as the negative control.

Isolates BT1, BT2, BT4, BT6, BT8 and BT9 showed significant degradation of *N*-hexanoyl homoserine lactone (C6-HSL); only trace or hardly any amounts of C6-HSL were detected by the biosensor CV026 (Figure 1). When tested for their ability to degrade *N*-decanoyl-L-homoserine lactone (C10-HSL), *N*-oxododecanoyl-L-homoserine lactone (3-oxo-C12-HSL) and pC-HSL, which represent long chain, oxo group substituted and aroyl group AHL substrates, respectively, they were also able to degrade the tested AHLs (Figures 2–4).

Results from whole-cell AHL inactivation assay and RRLC analyses on degradation of various AHL substrates indicated strong QQ activity with broad substrate specificity among the isolates of the genera of *Pseudomonas, Bacillus* and *Arthrobacter*. Our data illustrated that these soil QQ isolates exhibited broad AHL inactivation activity regardless of the *N*-acyl side chain length and degree of saturation at C3 position of the AHL molecules.

The QQ properties of these isolates have been well documented. For instance, AiiA lactonase homologs of *Bacillus*, AhlD lactonase of *Arthrobacter* and PvdQ and QuiP of *Pseudomonas* [31] have been well characterized. *Arthrobacter* is considered as one of the major groups among aerobic soil bacteria [32] but surprisingly, our previous effort did not isolate any *Arthrobacter* from Malaysian rain forest soil [22]. In this work, *Arthrobacter* isolate BT6 was isolated from Malaysian montane forest soil. This may be due to the cooler climate of this montane environment, in contrast to the warmer climate of our previous sampling site which was soil at the sea level. Aside from QQ activity, assessment of various biocatalytic tests on *Arthrobacter* sp. (BT6) also revealed positive results in proteolytic activity against casein and DNase activity (data not shown). It has been reported that members of *Arthrobacter* have diverse metabolic traits and are capable of degrading aromatic and aliphatic compounds including synthetic compounds such as pesticides [4,33].

A recent work by Momb [34] described the QQ activity of AiiA homolog from *Bacillus thuringiensis* against aroyl HSL. The *Arthrobacter* and *Pseudomonas* isolates in this study were also found to degrade pC-HSL. However, the mechanism of degradation of pC-HSL by these isolates remains unknown.

### 3.3. Identification of AHL Production

**Figure 5.**
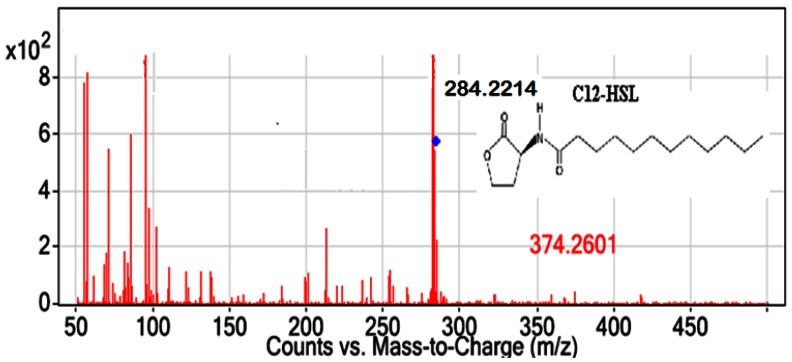
ESI-MS spectrum of C12-HSL (*m/z* 284.2214, 7.708 min).

All *Pseudomonas* isolates, but not the Gram positive isolates, triggered CV026 violacein production suggesting production of short chain AHL (data not shown). The spent culture supernatants of QS bacterial isolates were analyzed with the Agilent 6500 Q-TOF LC/MS system. Mass spectrometry analysis of the supernatant of *P. frederiksbergensis* isolate BT9 confirmed the presence of *N*-dodecanoyl-L-homoserine lactone (C12-HSL) (*m/z* value of 284.2214) (Figure 5). However, no AHL molecules were detected from extracts of other isolates using MS analysis although these isolates triggered CV026 violacein production. The failure of LC/MS to detect short chain AHLs may be due to the QQ activity among these isolates. It is speculated that short chain AHLs were self-degraded by the *Pseudomonas* isolates at the time point when the supernatant was processed for LC/MS analysis. It is also speculated that the amount of AHL molecules being produced was below the detection limit of the instrument even though detection of synthetic short chain AHL was feasible in this experiment. Alternatively, detection of short chain AHL could be accomplished by using gas chromatography mass spectrometry analysis as suggested by Cataldi and co-workers [35]. As existence of diverse QS systems and high complexity of QS regulation are common in *Pseudomonas* [36], our group is currently engaging whole genome sequence of these isolates as a means to gain a clearer view on the global regulation of the QS regulated genes.

## 4. Conclusions

We report here the degradation of AHLs by *Pseudomonas, Bacillus* and *Arthrobacter* isolated from Malaysian tropical montane soil. Our data demonstrated that six out of nine isolates studied significantly degraded C6-HSL and showed broad AHL inactivation specificity towards different AHL molecules. Long chain AHL namely C12-HSL was detected in the spent supernatant of *P. frederiksbergensis* isolate BT9, which also possessed strong QQ activity. Further investigations are being carried out to confirm the QS- and QQ-genes of *P. frederiksbergensis* isolate BT9.

## Figures and Tables

**Table 1.** Bacterial strains used.

Bacterial strain	Description	Source/Reference
*Chromobacterium violaceum* CV026	Mini-Tn*5* mutant derived from *C. violaceum* ATCC 31532 acts as biosensor with formation of purple violacein pigment in the presence of short chain exogenous AHL molecules.	[23]
*Erwinia carotovora* Attn	Positive control for QS tests owing to its capability of producing AHL molecules to be detected by *C. violaceum* CV026.	Gift from Prof. Paul Williams
*Erwinia carotovora* A20	Negative control for AHL production as it does not produce AHL molecules.	Gift from Prof. Paul Williams
*Bacillus cereus*	Positive control for QQ tests owing to its strong QQ activity.	[22]
*Escherichia coli* TOP10	Negative control for QQ tests as it does not possess QQ ability. It was also used as a host for TA cloning.	Invitrogen
Montane soil isolates BT1-BT9	This study	Cameron Highlands, Malaysia

**Table 2.** Identification of bacterial isolates and their respective accession numbers acquired from GenBank.

Strain	Identification	Accession Number after Sequence Deposition

BT1	*Bacillus* sp.	JN695052
BT2	*Bacillus* sp.	JN695053
BT3	*Pseudomonas frederiksbergensis*	JN695055
BT4	*Bacillus cereus*	JN695054
BT5	*Pseudomonas frederiksbergensis*	JN695056
BT6	*Arthrobacter* sp.	JQ014617
BT7	*Pseudomonas frederiksbergensis*	JN695057
BT8	*Pseudomonas frederiksbergensis*	JN695058
BT9	*Pseudomonas frederiksbergensis*	JN695059

## References

[1] González J.E., Marketon M.M. (2003). Quorum sensing in nitrogen fixing rhizobia. Microbiol. Mol. Biol. Rev..

[2] Hong K.W., Koh C.L., Sam C.K., Yin W.F., Chan K.G. (2012). Quorum quenching revisited—From signal decay to signalling confusion. Sensors.

[3] Fuqua C., Parsek M.R., Greenberg E.P. (2001). Regulation of gene expression by cell-to-cell communication: Acyl-homoserine lactone quorum sensing. Annu. Rev. Genet..

[4] Williams P., Winzer K., Chan W., Cámara M. (2007). Look who's talking: Communication and quorum sensing in the bacterial world. Phil. Trans. R. Soc. B..

[5] Swift S., Karlyshev A.V., Fish L., Durant E.L., Winson M.K., Chhabra S.R., Williams P., Macintyre S., Stewart G.S.A.B. (1997). Quorum sensing in *Aeromonas hydrophila* and *Aeromonas salmonicida*: Identification of the LuxRI homologs AhyRI and AsaRI and their cognate *N*-acylhomoserine lactone signal molecules. J. Bacteriol..

[6] Parsek M.R., Greenberg E.P. (2000). Acyl-homoserine lactone quorum sensing in Gram-negative bacteria: A signaling mechanism involved in association with higher organisms. Proc. Natl. Acad. Sci. USA.

[7] Dong Y.H., Gusti A.R., Zhang Q., Xu J.L., Zhang L.H. (2002). Identification of quorum quenching *N*-acyl homoserine lactonases from *Bacillus* species. Appl. Environ. Microbiol..

[8] Oger P., Kim K.S., Sackett R.L., Piper K.R., Farrand S.K. (1998). Octopine-type Ti plasmids code for a mannopine-inducible dominant-negative allele of *traR*, the quorum-sensing activator that regulates Ti plasmid conjugal transfer. Mol. Biol..

[9] Zhu J., Winans S.C. (1998). Activity of the quorum-sensing regulator *traR* of *Agrobacterium tumefaciens* is inhibited by a truncated, dominant defective *traR*-like protein. Mol. Microbiol..

[10] Glessner A., Smith R.S., Iglewski B.H., Robinson J.B. (1999). Roles of *Pseudomonas aeruginosa las* and *rhl* quorum-sensing systems in control of twitching motility. J. Bacteriol..

[11] Schaefer A.L., Greenberg E.P., Oliver C.M., Oda Y., Huang J.J., Banin G.B., Peres C.M., Schmidt S., Juhaszova K., Sufrin J.R., Harwood C.S. (2008). A new class of homoserine lactone quorum sensing signals. Nature.

[12] Lin Y.H., Xu J.L., Hu J., Wang L.H., Ong S.L., Leadbetter J.R., Zhang L.H. (2003). Acyl-homoserine lactone acylase from *Ralstonia* strain XJ12B represents a novel and potent class of quorum-quenching enzymes. Mol. Microbiol..

[13] Dong Y.H., Wang L.H., Zhang L.H. (2007). Quorum-quenching microbial infections: Mechanisms and implications. Phil. Trans. R. Soc. B.

[14] De Kievit T.R., Iglewski B.H. (2000). Bacterial quorum sensing in pathogenic relationships. *Infect. Immun*.

[15] Sio C.F., Otten L.G., Cool R.H., Diggle S.P., Braun P.G., Bos R., Daykin M., Cámara M., Williams P., Quax W.J. (2006). Quorum quenching by an *N-*acyl-homoserine lactone acylase from *Pseudomonas aeruginosa* PAO1. Infect. Immun..

[16] Wang Y.J., Leadbetter J.R. (2005). Rapid acyl-homoserine lactone quorum signal biodegradation in diverse soil. Appl. Environ. Microbiol..

[17] Uroz S., Chhabra S.R., Cámara M., Williams P., Oger P., Dessaux Y. (2005). *N*-acylhomoserine lactone quorum sensing molecules are modified and degraded by *Rhodococcus erythropolis* W2 by both amidolytic and novel oxidoreductase activities. Microbiology.

[18] Chen X., Schauder S., Potier N., Dorsselaer A.V., Pelczer I., Bassler B.L., Hughson F.M. (2002). Structural identification of a bacterial quorum sensing signal containing boron. Nature.

[19] Chong Y.M., Yin W.F., Ho C.Y., Mustafa M.R., Hadi A.H.A., Awang K., Narrima P., Koh C.L., Appleton D.R., Chan K.G. (2011). Malabaricone C from *Myristica cinnamomea* exhibits anti-quorum sensing activity. J. Nat. Prod..

[20] Hentzer M., Wu H., Anderson J.B., Riedel K., Rasmussen T.B., Bagge N., Kumar N., Schembri M.A., Song Z., Kristofferson P., Manfield M., Costerton J.W., Molin S., Eberl L., Steinberg P., Kjelleberg S., Hoiby N., Givskov M. (2003). Attenuation of *Pseudomonas aeruginosa* virulence by quorum sensing inhibitors. EMBO. J..

[21] Ulrich R.L. (2004). Quorum quenching: Enzymatic disruption of *N*-acylhomoserine lactose-mediated bacterial communication in *Burkholderia thailandensis*. Appl. Environ. Microbiol..

[22] Chan K.G., Wong C.S., Yin W.F., Sam C.K., Koh C.L. (2010). Rapid degradation of *N*-3-oxo-acylhomoserine lactones by a *Bacillus cereus* isolate from Malaysian rainforest soil. Antonie van Leeuwenhoek.

[23] McClean K.H., Winson M.K., Fish L., Taylor A., Chhabra S.R., Cámara M., Daykin M., Lamb J.H., Swift S., Brcroft B.W., Stewart G.S., Williams P. (1997). Quorum sensing and *Chromobacterium violaceum*: Exploitation of violacein production and inhibition for the detection of *N*-acylhomoserine lactones. Microbiology.

[24] Chan K.G., Yin W.F., Sam C.K., Koh C.L. (2009). A novel medium for the isolation of *N-*acylhomoserine lactone degrading bacteria. J. Ind. Microbiol. Biotechnol..

[25] Tamura K., Peterson D., Stacher G., Nei M., Kumar S. (2011). MEGA5: Molecular evolutionary genetics analysis using maximum likelihood, evolutionary distance, and maximum parsimony methods. Mol. Biol. Evol..

[26] Chan K.G., Atkinson S., Mathee K., Sam C.K., Chhabra S.R., Koh C.L., Williams P. (2011). Characterization of *N*-acylhomoserine lactone-degrading bacteria associated with the *Zingiber offinale* (ginger) rhizosphere: Co-existence of quorum quenching and quorum sensing in *Acinetobacter* and *Burkholderia*. BMC Microbiol.

[27] Wong C.S., Yin W.F., Choo Y.M., Sam C.K., Koh C.L., Chan K.G. (2011). Coexistence of quorum-quenching and quorum-sensing in tropical marine *Pseudomonas aeruginosa* strain MW3A. World J. Microbiol. Biotechnol..

[28] Shirai Y. (2000). Temperature tolerance of diamondback moth, *Plutella xylostella* (Lepidoptera: Yponomeutidae) in temperate and tropical regions of Asia. Bull. Entomol. Res..

[29] Schweizer P., Schlagenhauf E., Schaffrath U., Dudler R. (1999). Different patterns of host genes are induced in rice by *Pseudomonas syringae*, a biological inducer of resistance and the chemical inducer Benzothiadiazole (BTH). Eur. J. Plant. Pathol..

[30] Setlow P. (2006). Spores of *Bacillus subtilis*: Their resistance to and killing by radiation, heat and chemicals. J. Appl. Microbiol..

[31] Dong Y.H., Zhang L.H. (2005). Quorum sensing and quorum-quenching enzymes. J. Microbiol..

[32] Park S.Y., Lee S.J., Oh T.K., Oh J.W., Koo B.T., Yum B.T., Lee J.K. (2003). AhlD, an *N*-acylhomoserine lactonase in *Arthrobacter* sp., and predicted homologues in other bacteria. Microbiology.

[33] Keuth S., Rehm H.J. (1991). Biodegradation of phenanthrene *by Arthrobacter polychromogenes* isolated from a contaminated soil. *Appl. Microbiol. Biotech*.

[34] Momb J., Yoon D.W., Fast W. (2010). Enzymic disruption of *N*-aroyl-L-homoserine lactone-based quorum sensing. ChemBioChem.

[35] Cataldi T.R.I., Bianco G., Frommberger M., Schmitt-Kopplin P. (2004). Direct analysis of *N*-acyl-L-homoserine lactone using gas chromatography/mass spectrometry. Rapid Commun. Mass Spectrom..

[36] Venturri V. (2006). Regulation of quorum sensing in *Pseudomonas*. FEMS Microbiol. Rev..

